# C/EBP Homologous Protein Expression in Retinal Ganglion Cells Induces Neurodegeneration in Mice

**DOI:** 10.3390/ijms26051858

**Published:** 2025-02-21

**Authors:** William C. Mayhew, Balasankara Reddy Kaipa, Linya Li, Prabhavathi Maddineni, Yogapriya Sundaresan, Abbot F. Clark, Gulab S. Zode

**Affiliations:** 1 North Texas Eye Research Institute, Department of Pharmacology and Neuroscience, University of North Texas Health Science Center, Fort Worth, TX 76107, USA; williammayhew@my.unthsc.edu (W.C.M.); abe.clark@unthsc.edu (A.F.C.); 2 Gavin Herbert Eye Institute-Center for Translational Vision Research, Department of Ophthalmology, University of California Irvine School of Medicine, Irvine, CA 92697, USA; bkaipa@hs.uci.edu (B.R.K.); linyal1@hs.uci.edu (L.L.); ysundare@hs.uci.edu (Y.S.); 3 Department of Ophthalmology, School of Medicine, University of Missouri, Columbia, MO 65201, USA; pmbnv@health.missouri.edu

**Keywords:** glaucoma, ER stress, CHOP, ATF4, neurodegeneration, retinal ganglion cells

## Abstract

The progressive loss of retinal ganglion cell (RGC) axons leading to irreversible loss of vision is the pathological hallmark of glaucoma. However, the pathological mechanisms of RGC degeneration are not completely understood. Here, we investigated the role of chronic endoplasmic reticulum (ER) stress in glaucomatous neurodegeneration. To evaluate whether chronic ER stress-induced transcriptional factors, activating transcription factor 4 (ATF4), and C/EBP homologous protein (CHOP) are induced in RGCs; we utilized human donor tissue and the microbead occlusion model of glaucoma. Additionally, we performed the intravitreal injection of adeno-associated virus (AAV) 2 to express CHOP selectively in RGCs in C57BL/6 mice and evaluated its effect on RGC function and structure by pattern electroretinogram (PERG) and whole-mount retina staining with the RBPMS antibody. Here, we report that the ATF4-CHOP pathway is activated in the retinas of human glaucoma donor eyes and a mouse model of ocular hypertension. Further, the expression of CHOP in RGCs led to a significant loss of function, as evidenced by reduced PERG. Notably, the expression of CHOP in the retina induced a significant structural loss of RGCs within 15 weeks of injection. Altogether, our studies indicate that the expression of CHOP in RGCs leads to neurodegeneration in mice.

## 1. Introduction

Glaucoma is a group of multifactorial eye diseases characterized by progressive optic nerve degeneration and vision loss. Primary open-angle glaucoma (POAG) is the most common form of glaucoma [1], affecting over 70 million of the global population; it is projected to reach 111.8 million by 2040 [2,3]. Elevated intraocular pressure (IOP) is the only modifiable risk factor for POAG. Current treatments for POAG include IOP-lowering pharmaceuticals and surgeries [4,5]. Some patients are not responsive to IOP-lowering drugs and continue to lose their vision [6]. In a subset of POAG patients, vision loss occurs despite normal IOP [7,8]. Therefore, it is critical to understand the pathological mechanisms of glaucomatous neurodegeneration and develop targeted neuroprotective therapies to prevent vision loss.

Endoplasmic reticulum (ER) stress has been shown to play an important role in the development of several neurodegenerative diseases, including glaucoma [9,10,11,12]. The ER is responsible for much of the cell’s synthesis and folding of proteins. Additionally, the ER is a critical sensor and regulator for stress within the cell via the autophagy and neuroinflammation pathways. The aggregation of misfolded proteins in the ER can overload the cell and impair its ability to regulate homeostasis. ER stress induces the unfolded protein response (UPR) pathway, which works to reduce protein synthesis and clear aggregates within the cell [13,14,15]. Misfolded proteins are sensed by the ER stress sensors PERK, ATF6α, and IRE1, activating the protective branches of the UPR pathway to remediate aberrant protein synthesis and normalize ER homeostasis [11,16]. Activated PERK phosphorylates eIF2α, which drastically decreases protein translation and induces the expression of the transcription regulator protein, activating transcription factor 4 (ATF4). The activation of ATF4 triggers the expression of several ER stress-associated proteins, including CCAAT-enhancer-binding protein–homologous protein (CHOP). Once CHOP is expressed, it induces the phosphatase GADD34, which dephosphorylates elF2α and reverses the inhibition of protein translation. Phosphorylated IRE1 converts XBP-1 into the spliced form, XBP-1s, which initiates the transcription of genes that encode the glucose-regulated ER stress chaperones GRP78 and GRP94 and the ER-associated degradation (ERAD) pathway [11,17]. Activated ATF6 also controls the expression of certain UPR genes to resolve ER stress [16,18,19]. With chronic ER stress, the UPR can shift from a cytoprotective repair pathway to an apoptotic pathway, resulting in cell death [20,21,22]. Though the precise molecular mechanism is unknown, there is a clear link between the PERK-ATF4-CHOP signaling and ER stress-induced apoptosis [20,23,24,25,26].

RGC degeneration is a key feature of glaucomatous neurodegeneration. Previous studies have shown that ER stress markers, including CHOP, are induced in a mouse model of acute IOP elevation and an optic nerve crush model [11]. IOP elevation from laser photocoagulation in the eyes of cynomolgus monkeys induced CHOP and led to neuronal loss in the lateral geniculate nucleus region of the brain [27]. The deletion of CHOP and the activation of XBP-1 increased RGC survival in optic nerve crush and the microbead-occlusion model of ocular hypertension [10]. The elevation of IOP in rats by episcleral vein occlusion causes CHOP elevation, TUNEL-positive cells in the ganglion cell layer (GCL), and a decrease in RGC count [28]. In normal-tension glaucoma, an optineurin polymorphism (M98K) has been shown to make RGCs more susceptible to ER stress and TNFα-associated cell death pathways [29]. While there are many known characteristics and links between glaucomatous neurodegeneration and the ER stress pathway, the initiating trigger and precise mechanisms of action of RGC dysfunction/death are not yet fully understood. Specifically, it is not understood whether the induction of ER stress alone is sufficient to cause neurodegeneration. Here, we propose that IOP elevation induces chronic ER stress-associated pro-apoptotic transcriptional factor, ATF4, and CHOP in RGCs, leading to neurodegeneration. To test our hypothesis, we examined whether ATF4 and CHOP are induced in RGCs of glaucoma donor eyes and a mouse model of ocular hypertension. Our studies demonstrate that the induction of CHOP in RGCs is associated with glaucomatous neurodegeneration. We further determined that forced expression of pro-apoptotic CHOP in RGCs is sufficient to induce neurodegeneration in mice.

## 2. Results

**Figure 1:**
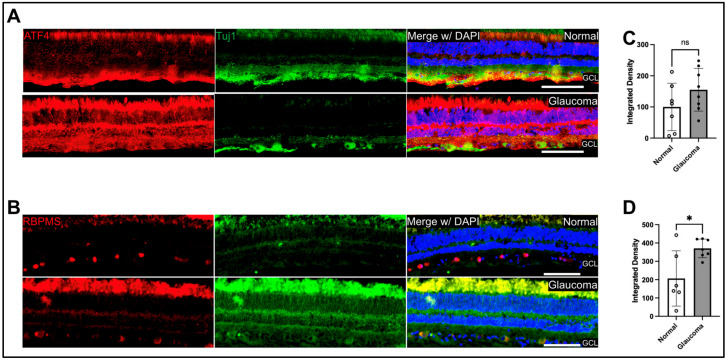
Immunohistochemical analysis of human retinal cross-sections of ER stress markers, ATF4, and CHOP. Representative immunostaining images for (**A**) ATF4 and (**B**) CHOP in normal and glaucoma donor retinas. The scale bar is 100 μm. GCL-integrated density measurements for (**C**) ATF4 were not statistically significant using an unpaired *t*-test with an n of 7–8 retinas for normal and glaucoma, respectively. (**D**) CHOP in normal and glaucoma retinas revealed that CHOP is significantly increased (* *p* < 0.05) in glaucoma donor eyes with n of 8 retinas for normal and glaucoma, using an unpaired *t*-test. Error bars are displayed as SD of the mean.

**Increased CHOP in RGCs of glaucoma donor eyes:** To evaluate whether the chronic ER stress markers are increased in glaucomatous RGCs, we performed immunohistochemical analysis on sagittal sections of paraffin-embedded human donor retinas from glaucomatous and healthy control eyes (Figure 1) (Supplemental Figure S1). ATF4 protein levels appeared to be increased in the ganglion layer at the periphery of glaucomatous human retinas, but it was not statistically significant compared to control retina (n = 8). CHOP was significantly increased in the ganglion cell layer in the peripheral region of glaucomatous retinas (n = 7; *p* < 0.05). CHOP expression in the other retinal regions appeared to increase but was not statistically significant.

**Figure 2:**
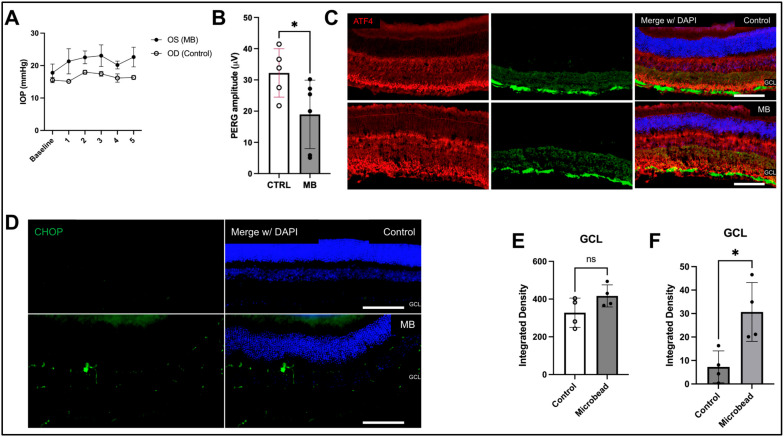
ATF4 and CHOP are induced in a mouse model of ocular hypertension. (**A**) IOP measurements of microbead (MB)-injected mice compared to control mice 5 weeks post-injection (n = 6). Error bars are displayed as SEM. (**B**) PERG analysis showing a functional loss of RGCs in the MB-injected mice (n = 6), compared to controls (n = 5). Representative images of MB and control retinal sections stained with (**C**) ATF4 and neuronal marker β-tubulin III (Tuj1) or (**D**) CHOP and RBPMS Scale bar is 100 μm. Integrated densities of (**E**) ATF4 were not statistically significant using an unpaired *t*-test (n = 4) for control and MB, respectively, and (**F**) CHOP in control and MB-injected mice showed significantly increased CHOP (* *p* < 0.01) in RGCs of MB-injected mice (n = 4), unpaired *t*-test.

**ATF4 and CHOP are induced in RGCs of ocular hypertensive mice:** The magnetic microbead occlusion model (MB) was performed as previously described [30,31]. As shown in Figure 2A,B, intracameral injections of microbeads led to significant IOP elevation and induced a functional loss of RGCs. We observed consistent IOP elevation in about 60–70% of eyes injected with MB compared to control-injected eyes. IOP increases of 4 mmHg or higher over 5 weeks were followed with PERG to ensure the functional loss of RGCs (Figure 2B). One of the control eyes was removed from the dataset due to amplitude values that were outside of the expected range for PERG amplitude in mice. Retinal tissues were collected at this stage and analyzed for ER stress markers (Figure 2C,D) (Supplemental Figure S2). CHOP is significantly induced in the RGCs of ocular hypertensive mice injected with MB (Figure 2F). We also observed that ATF4 staining appeared to be increased in the RGCs of MB-injected eyes, but the effect was not found to be statistically significant (Figure 2E).

**Figure 3:**
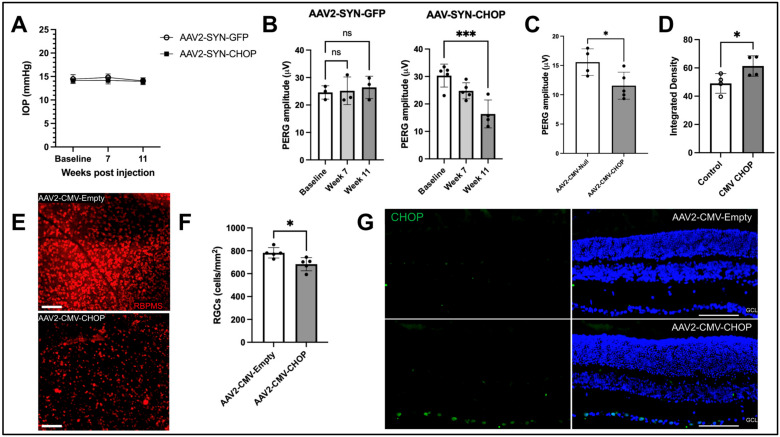
Expression of CHOP in RGCs leads to neurodegeneration in mice. Two-month-old C57BL6/2J were injected intravitreally with AAV2-Empty or CHOP, and IOPs, PERG, and RGC loss were analyzed. (**A**) IOP measurements showed no change in IOP between control and AAV2-SYN-CHOP injected mice (n = 4). Error bars are displayed as SEM. (**B**) PERG analysis showing significantly reduced amplitudes (*** *p* < 0.001) in 11-week AAV2-SYN-CHOP-injected mice compared to AAV2-SYN-Empty-injected controls using a two-way ANOVA comparing subsequent measurements to the baseline with an n of 3–5 for controls and AAV2-SYN-CHOP, respectively. Error bars are displayed as SD of the mean. (**C**) PERG analysis of AAV2-CMV-CHOP showing a significant decrease from empty control vector (* *p* < 0.05) using an unpaired *t*-test (n = 4, control) (n = 5, CHOP). (**E**,**F**) Whole mount retina staining of AAV2-CMV-CHOP mouse retinas with RBPMS antibody showing significant loss of RGCs in 15-week CHOP-expressed retinas compared to the controls. Images represent extreme RGC loss observed in some retinas. RGC counts were analyzed with an unpaired *t*-test (n = 5) (* *p* < 0.05). Error bars are displayed as SD of the mean. (**D**) Integrated density of CHOP immunofluorescent staining in the GCL of AAV2-CMV-CHOP mouse retinas, compared to control retinas (n = 4) (* *p* < 0.05). (**G**) Representative staining of mouse retinas from AAV2-CMV-Empty or CHOP-injected mice showing CHOP expression in RGCs of AAV2-CMV-CHOP retinal cross-sections. The scale bar is 100 μm.

**Figure 4:**
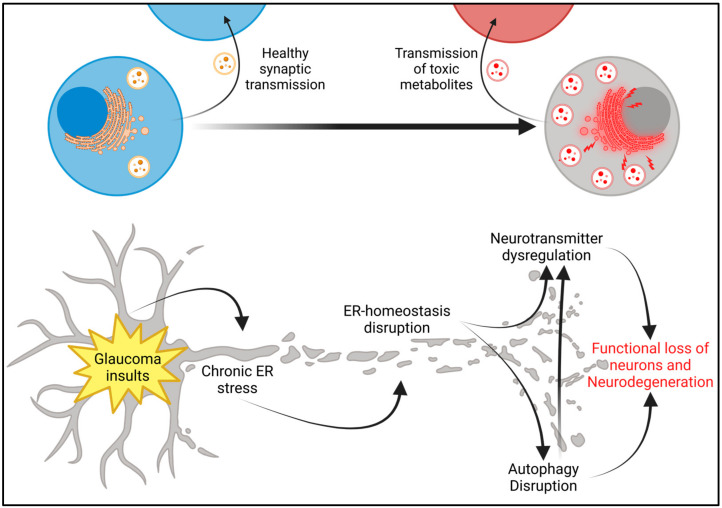
The proposed hypothesis. Glaucomatous insults to the retina trigger a cascade that starts with the induction of ER stress and disruption of ER homeostasis. This impairs autophagy and accumulates toxic aggregates that cause dysfunction and neurodegeneration of the RGCs and are potentially transmitted to other neurons in the retina.

**Expression of CHOP in RGCs leads to neurodegeneration:** Since CHOP is induced in RGC axons of mouse and human glaucoma, we next examined whether the expression of CHOP in RGCs leads to neurodegeneration. C57BL/6J mice were intravitreally injected with AAV2 expressing either CHOP or an empty viral package under the control of the human synapsin (SYN) promoter. We observed that the expression of CHOP did not alter IOPs significantly (Figure 3A), and IOPs were similar in both empty and CHOP-expressing mice. Next, we examined the RGC function using PERG measurements 7 and 10 weeks after injections of the AAV2-SYN vector (Figure 3B) and AAV2-CMV vectors (Figure 3C). As expected, the empty vector did not alter PERG amplitude significantly. Expression of AAV2-SYN-CHOP did not significantly alter PERG amplitudes at 7 weeks of injection but reduced PERG amplitude significantly (by 50%) 11 weeks post-injection (Figure 3B). We next examined whether the expression of CHOP in RGCs leads to degeneration by performing whole-mount retina staining with RBPMS antibody. Interestingly, the expression of CHOP under the SYN promotor was not significantly different from controls. We repeated the experiment using the more robust CMV promotor to express CHOP at a higher level in the retina and found a significant decrease in RGCs in the peripheral retinas of CHOP-induced mice (Figure 3E,F). Representative immunostaining of RBPMS in the retina’s periphery (Figure 3E) and its analysis (Figure 3F) demonstrated that the expression of CHOP under the CMV promoter leads to significant loss of RGCs in the retina’s periphery. We did not observe any significant loss of RGCs in other geographic regions of the retina. We next evaluated CHOP expression in the RGCs after injection of AAV2-CMV-CHOP. AAV2-CMV-Empty or AAV2-CMV-CHOP-injected retinal cross-sections were stained with CHOP antibody, which showed clear CHOP induction in the GCL of the CHOP-expressed retinas compared to AAV2-Empty-injected retinas (Figure 3D,G) (Supplemental Figure S3). This shows that the induction of ER stress in the retina leads to the functional loss and progressive death of RGCs in glaucoma. We therefore propose that glaucomatous damage induces ER stress in RGCs and dysrupts ER homeostasis and leads to neuronal loss in glaucoma (Figure 4).

## 3. Discussion

Chronic ER stress is associated with disease pathology in several neurodegenerative diseases, including glaucoma [32,33,34,35]. Previous studies in our laboratory showing the role of ER stress in glaucoma have been conducted in the trabecular meshwork, where it causes the dysregulation of the aqueous humor outflow pathway, increasing the IOP in mouse models and human glaucoma [20,24,25,36,37]. Though ER stress is deeply associated with neurodegeneration, it is not known whether chronic ER stress alone is sufficient to cause neurodegeneration. Here, we show that RGCs have activated ATF4-CHOP signaling in human glaucoma and in response to ocular hypertension in mice. Additionally, forced expression of CHOP alone is sufficient to cause a functional loss of RGCs in mice.

Although both ATF4 and CHOP appeared to increase in the RGCs of glaucoma donor eyes (Figure 1A,B), we observed that immunostaining for CHOP but not ATF4 was significantly elevated in the RGCs of human glaucoma patients (Figure 1C,D). As the human eyes come from deceased patients outside of the control of a research study, it is important to note that many of the donors were in an advanced stage of glaucoma and had been taking multiple drugs to manage the disease. It is possible that these factors could influence ER stress. However, our findings in mice correspond with our human data, so we believe that if there is an effect caused by the advanced disease state or IOP-lowering medications, the effect is not significant enough to affect the results of this study. The immunostaining for ATF4 has a nonsignificant trend increase. This could be due to higher basal expression of ATF4 in the healthy cells compared to CHOP. Additionally, one study found that while CHOP mRNA increased, ATF4 mRNA remained the same after exposure to the ER stressor thapsigargin [38]. This could indicate that ATF4 upregulation is not required for the downstream pathway once CHOP has been induced. Another possibility is that CHOP is induced directly in RGCs, which are more susceptible to ER stress due to their high energy demand for the processing and transporting neurotransmitters for cell signaling [39,40,41]. More research is required to fully understand the dynamics of ATF4 and CHOP signaling in glaucomatous retinas and ER stress’s role in glaucomatous retinal degeneration.

Here, we report that ER stress markers are elevated in the magnetic microbead occlusion mouse model of ocular hypertension. This is a well-accepted model for studying the direct effect of ocular hypertension on RGCs [42,43]. As expected, the microbead model produced a sustained elevation of IOP and loss of RGC function as recorded by PERG. After six weeks of IOP elevation, we found that CHOP immunostaining significantly increased in the GCL of the mouse eyes that were treated with microbeads. As with our findings in human eyes, we detected a nonsignificant trend increase in ATF4 immunostaining in the microbead eyes compared to the controls. These results support our findings in human eyes and further indicate that ER stress plays a key role in RGC neurodegeneration in glaucoma.

Previously, we have reported that the overexpression of ATF4 but not CHOP alone in the TM causes TM dysfunction and IOP elevation [20]. However, CHOP was required to induce ATF4-mediated TM dysfunction. We also showed that ATF4 interacts with CHOP to induce TM dysfunction and IOP elevation, and CHOP was required for ATF4-mediated TM dysfunction. In this study, we report that the expression of CHOP alone in RGCs is sufficient to induce neurodegeneration. It is, therefore, plausible that CHOP plays a direct, pro-apoptotic role in glaucomatous neurodegeneration. Consistent with this, RBPMS staining demonstrated a significant RGC loss in the peripheral retinas of CHOP-overexpressed mice using the CMV promoter compared to empty controls. However, we did not observe a significant loss of RGCs in mice injected with AAV2-CHOP under the control of the human synapsin promoter. This discrepancy could be due to the human synapsin promoter having a robust enough expression of CHOP to cause neuronal dysfunction but not the death of RGCs in the mice. These findings show that the targeted induction of CHOP in the RGCs of mice is enough to cause a significant functional loss of RGCs in mice, independent of IOP.

The ER is responsible for significant portions of cell signaling. The smooth ER controls much of the synthesis of the membranes for vesicle transport of neurotransmitter precursors prior to packaging and modification by the Golgi [44]. The ER and mitochondria work together to release and sequester calcium to control cell signaling. Neurons rely heavily on tightly regulated calcium signaling and neurotransmitter production, making them highly susceptible to disruptions in ER homeostasis [41,45,46]. It has been established that the impaired autophagy of mitochondria can cause mitochondrial dysfunction and oxidative stress. Autophagy dysfunction has been linked to the pathology of many neurodegenerative disorders [47]. In Parkinson’s Disease, it has been shown that intermediates of dopamine synthesis play an integral role in the formation of Lewy bodies, the hallmark protein aggregates observed in the disease [48,49]. We, therefore, propose that forced induction of CHOP in the retinas of mice might impair autophagy and lead to neurotransmitter dysregulation. This could lead to neuronal dysfunction, cell death, and a decreased capacity for mitochondrial turnover. While GCL seemed to be the common denominator for ER stress in our study, there appeared to be additional cells in other retinal layers that may also be affected by ER stress. It has been shown that photoreceptors may also be affected by glaucomatous insults in human glaucoma patients and Rhesus monkeys with laser-induced IOP elevation [50]. Additionally, in laser-induced IOP elevation in rats, cone photoreceptors were significantly decreased compared with controls [51]. It is possible that the expression of CHOP in RGCs leads to the accumulation of proteins and organelles in the RGC soma and synapses due to defective autophagy, which may lead to the synaptic transmission of aggregated signaling molecules and neurotransmitters from RGCs to neurons in other retinal layers. Additionally, there is evidence that retinal astrocytes and microglia may propagate ER stress in neurodegenerative diseases through inflammation, providing another mechanism by which a cellular dysfunction can lead to tissue damage in a localized area [52]. In this study, we limited our scope to investigate the effect of ER stress on RGCs. Further studies will be required to thoroughly understand the mechanism by which ER stress leads to glaucomatous neurodegeneration in the retina.

Similar patterns of ER stress-induced neuronal dysfunction and degeneration have also been observed in other neurodegenerative diseases, suggesting a possible shared mechanism of neurodegeneration among diseases [13,21,32,33,53,54,55,56]. Hu and colleagues found that CHOP deletion increased RGC survival in axotomized optic nerves [11]. Though ER stress is associated with glaucoma and the modulation of CHOP reduces damage to RGCs following insult, the mechanism by which ER stress leads to RGC dysfunction and death is not known. In this paper, we described a pathway by which ER stress in the retina causes RGC dysfunction and cell death in POAG. If ER stress is a common pathway of neurodegeneration, this discovery could increase our knowledge of retinal glaucoma pathology and add to our understanding of the general process of neurodegeneration across other diseases. Importantly, these findings suggest that targeting mitophagy in the retina can be a promising strategy for treating glaucomatous neurodegeneration.

In summary, we report that the ATF4-CHOP ER stress pathway is activated in human glaucomatous neurodegeneration and a mouse model of ocular hypertension. The forced expression of CHOP leads to the loss of RGCs in mice. This study has shown that glaucomatous retinal degeneration shares many facets with other hallmark neurodegenerative diseases. This common degeneration pathway could prove to be a prime target for neuroprotective therapies for glaucoma and other diseases that share UPR dysfunction. Importantly, our studies suggest that targeting autophagy or chronic ER stress in the retina can be an attractive strategy to stop glaucomatous neurodegeneration.

## 4. Materials and Methods

**Antibodies and reagents:** The following viral vectors and reagents were utilized in our study, and the list of antibodies can be found in Table 1. AAV2-CMV-Empty, AAV2-CMV-CHOP, and AAV2-SYN-CHOP were purchased from Vector Builder, Inc. (Chicago, IL, USA) Goat serum (Millipore, S26) (Burlington, MA, USA), isoflurane (Covetrus, NDC: 116950-6777-2) (Portland, ME, USA), ketamine (Covetrus, NDC: 11695-0703-1), microbeads (Bangs Laboratories, UMFR003) (Fishers, IN, USA), mounting media with DAPI (Vectashield, H-1200-10) (Newark, CA, USA), mydriacyl (Alcon, NDC: 0998-0355-15) (Geneva, Switzerland), phosphate-buffered saline (PBS) (Genessee Scientific, 25-507) (Morrisville, NC, USA), proparacaine (Alcon, NDC: 0998-0016-15), Triton-X100 (Millipore, TX8787), and xylazine (Covetrus, NDC: 59399-110-20).

**Human Donor Eyes:** Studies involving human tissue were performed in accordance with the guidance of the Declaration of Helsinki. Donor eyes were acquired with informed consent from the Lions Eye Institute for Transplant and Research (now the Lions World Vision Institute, Tampa, FL, USA). Donor eyes were processed within 24 h post-mortem. The donor information is provided in Table 2.

**Animals:** Three-month-old C57B/6J mice were obtained from the Jackson laboratory (Stock # 000664) and kept in a 12 h/12 h light/dark cycle at the appropriate ambient humidity (40–70%) and temperature (21–24 °C). Animals had access to standard rodent chow and water ad libitum. All animal experiments were conducted according to the ARVO Statement for the Use of Animals in Ophthalmic and Visual Research and were approved by the Institutional Animal Care and Use Committee (IACUC) of the University of North Texas Health Science Center (UNTHSC) (IACUC-2021-0036). Baseline measurements of IOP and pattern electroretinography (PERG) were performed prior to ocular hypertension or viral induction interventions. Topical antibiotics were applied after intraocular injections, and mice were removed from the isoflurane anesthesia nose cone and monitored until they awoke and became fully alert. Mice were euthanized by the inhalation of carbon dioxide followed by cervical dislocation.

**Microbead ocular hypertensive (OHT) model:** Mice were anesthetized by vaporized isoflurane (2.5%), and the eyes were treated with a topical analgesic, proparacaine HCl (0.5%) (Alcon: NDC, 0998-0016-15) and mydriacyl (1%) (Alcon, NDC: 0998-0355-15). In total, 2 µL of a suspension containing magnetic 8 µm microbeads (Bangs Laboratories: UMFR003) was injected intracamerally into the anterior chamber of the eye using a pulled glass micropipette and a manual micro syringe pump (World Precision Instruments: M3301-M3-R) (Sarasota, CA, USA) as described previously [30]. After the injection, but before removing the micropipette, a neodymium magnet was used to drag the beads around the limbus, ensuring adequate coverage of the trabecular meshwork and sufficient outflow blockage to develop ocular hypertension. IOPs were measured weekly to validate OHT, and PERG was measured at three weeks and six weeks to track the progression of retinal degeneration. After six weeks, mice were euthanized, and eyes along with optic nerves were fixed with 4% paraformaldehyde (PFA) and utilized for ER stress markers analysis.

**Viral transduction:** Baseline measurements of PERG and IOPs were performed before the viral transduction of the retina. The mice were anesthetized using vaporized isoflurane using a Kent scientific VetFlo vaporizer (Torrington, CT, USA), and a single drop of proparacaine HCl (0.5%) was applied to the eyes for topical anesthesia. Then, 2 µL of AAV2-Empty or CHOP, containing approximately 1.0 × 10^10^ genome copies, was injected intravitreally using a 10 µL syringe with a 33-gauge needle as described previously [20,57]. The mice were monitored weekly for changes in IOP and gross anatomical ocular changes via a dissection microscope (Leica: M205) (Tokyo, Japan). The mice were evaluated monthly for retinal function by measuring PERG.

**IOP:** Intraocular pressure was measured weekly, between 8:00 a.m. and 10:00 a.m. post-treatment, as described previously [24,26,36]. The mice were anesthetized with 2.5% vaporized isoflurane using a 0.8 L/min oxygen flow rate. A handheld tonometer (TonoLab: TV02) (Vantaa, Finland) was used to collect IOP measurements. Six measurements were averaged to obtain a single IOP value for each eye. Successful IOP elevation was characterized by a delta increase of 4 mmHg over the baseline IOPs.

**PERG**: Retinal function was evaluated by PERG as described previously [24,58]. Mice were anesthetized by intraperitoneal injection of a 100/10 mg/kg mixture of ketamine and xylazine. Anesthetized mice were placed on a temperature-controlled platform of the PERG machine (Jorvec: Miami, FL, USA), facing the signal transducing screens [59]. Measurements of the PERG amplitude and latency were collected in a dark room, using two measurements of 372 sweeps, as described previously [20,58]. Using the manufacturer’s recommended settings, PERG measurements for AAV2-CMV-injected mice were performed with the Celeris system (Diagnosys LLC, Lowell, MA, USA). PERG amplitudes were collected to assess RGC function as previously described [60].

**Paraffin embedding and sectioning:** The enucleated post-mortem human eyes were immersed in 4% PFA for 48 h. The anterior segment and vitreous gel were removed from human donor eyes, and the posterior cup was fixed again in 4% PFA in PBS for two hours at room temperature and washed in PBS. The posterior cup, including the optic nerve, was sagittally bisected. Each half of the posterior cup was placed in 70% ethanol overnight at 4 °C. The tissues were perfused with paraffin wax using a tissue processor (Tissue Tek VIP) (Torrance, CA, USA) and then embedded in paraffin wax blocks. Paraffin-embedded tissues were sectioned on a microtome (Leica RM 2255) (Tokyo, Japan) at 5 µm thickness and placed onto charged glass microscope slides.

Human retinal sections were dehydrated by heating in an oven at 60 °C for six hours. The slides were then deparaffinized by two washes of xylenes, and then gradient rehydration from 100% to 50% ethanol, before rinsed with PBS. The deparaffinized slides were then submerged in a citrate buffer (pH 6) and heated at 70 °C for 90 min for antigen retrieval. The permeabilized slides were then left to cool at room temperature for twenty minutes and washed with PBS. A barrier was drawn around each retinal section on the slide using a hydrophobic pen (Fisher Scientific: NC9204359) (Waltham, MA, USA). Sections were blocked for two hours at room temperature in PBS with 10% goat serum (Millipore) and 2% Triton X-100 (TX-100) (Sigma) (St. Louis, MO, USA). One slide was incubated without primary antibody and served as a negative control. The remaining slides received primary antibody diluted in blocking buffer and incubated overnight at 4 °C. Slides were washed with PBS and then incubated with fluorophore-conjugated secondary antibody diluted in blocking buffer for two hours at room temperature, under dark conditions. The slides were washed with PBS and dried with Kimwipes. A cover glass was applied to each slide using mounting media with DAPI (Vectashield: H-1200-10) (Newark, CA, USA), and the edges were sealed with clear nail polish. Mounted slides were left to dry overnight at 4 °C and imaged by confocal microscopy (Leica DMI8) (Tokyo, Japan) the following day at 200× magnification. Primary antibody negative control images acquired to ensure that signal was not a result of autofluorescence (Supplemental Figure S4) Fluorescence was analyzed in Image J by selecting individual retinal layers to measure the integrated density of the target protein in the ganglion cell layer.

**OCT embedding, sectioning, and immunostaining:** Mice were euthanized and perfused with 4% PFA as previously described [61]. Mice were decapitated, and skulls were exposed with a sagittal cut from the base of the skull to the snout. The skull cap was removed, exposing the brain, which was lifted to reveal the optic nerves. The optic nerves were cut at the optic chiasm, and eyes were carefully removed by separating the eye from the muscle and connective tissue in the orbital socket and freeing the optic nerve from the skull. Mouse eyes with the optic nerves were fixed in a solution of 4% PFA for thirty minutes, then washed with PBS. The eyes were cryoprotected through serial incubations in sucrose (10–30%) at 4 °C, two hours at 10%, overnight at 20%, and overnight at 30%. The eyes were embedded into tissue molds with optimal cutting temperature compound (OCT) (Sakura Finetek: 4583) (Tokyo, Japan), flash frozen with liquid nitrogen vapor, and then placed in the −80 °C freezer overnight. The embedded eyes were sagittally sectioned through the optic nerve in a cryosectioning station (Leica CM3050) (Tokyo, Japan) at a thickness of 10 µm and placed onto charged glass microscope slides.

Slides containing sections of mouse retinas and optic nerves were heated at 60 °C for one hour to encourage strong tissue adherence to the glass. The slides were washed with PBS, then submerged in a PBS-blocking buffer containing 10% goat serum and 0.2% TX-100 for two hours to block and permeabilize the tissue to expose antigens for staining. Slides were wiped dry, and a barrier was drawn around each retinal section on the slide using a hydrophobic pen. The primary antibodies were diluted in blocking buffer and incubated overnight at 4 °C in a humid, dark tray. The primary antibody solution was washed off with PBS, and slides were incubated with fluorophore-conjugated secondary antibodies diluted in blocking buffer for 90 min at room temperature under dark conditions. Slides were washed with PBS and wiped clean with a Kimwipe before adding mounting media with DAPI and a cover glass, sealed to the slide with clear nail polish. Slides were left to dry overnight at 4 °C and imaged by confocal microscopy (Leica DMI8) (Tokyo, Japan) the following day at 200× magnification. Primary antibody negative control images acquired to ensure that signal was not a result of autofluorescence (Supplemental Figures S5 and S6). Fluorescence intensities were analyzed in Image J by drawing a selection around individual retinal layers to measure the integrated density of the target protein in the ganglion cell layer.

**Whole mount RGC staining:** PFA-perfused mouse eyes were cleared of all muscle, fat, and connective tissue. The anterior chamber and lens were carefully removed, and Retinas were fixed in 4% PFA for 2 h. Four cuts were made to create four quadrants from the posterior cup. The retina was carefully detached from the sclera and placed in a well of a 48-well plate containing PBS. The retina was blocked with 10% goat serum and 0.2% TritonX-100 in PBS overnight at 4 °C. The blocking buffer was aspirated from the wells, and the RBPMS antibody was diluted in the blocking buffer and added to the retinas to incubate at 4 °C for three days. Retinas were thoroughly washed in PBS for three hours and then incubated with a goat-anti-rabbit AlexaFluor secondary antibody for three hours at room temperature. The retinas were washed in PBS for two hours at room temperature and placed onto a glass microscope slide RGC side up, and mounting media with DAPI and cover glass was added, followed by sealing with clear nail polish. Images were taken with confocal microscopy (Leica DMI8) (Tokyo, Japan) at 200× magnification and analyzed by an automated macro in ImageJ to count the RBPMS-positive cells in the retinas.

**IHC image analysis:** Images within the same comparison groups for all experiments were acquired on the same day, using the same microscope settings for laser intensity and detector gain. For human retinal analysis, integrated density was measured in the ganglion cell layer using ImageJ. Each data point displayed represents an average of three integrated density measurements per region per sample. For IHC analysis of mouse retinal cross-sections, integrated density was measured using ImageJ software version 1.54. Each biological data point was averaged from 11 to 18 images across 3 technical replicates of immunofluorescent staining of the same eye. Whole mount analysis of RBPMS staining comprised 24 images per eye. The geographic distribution of images was 12 peripheral, 8 mid-peripheral, and 4 central images.

## Figures and Tables

**Table 1:** Antibodies.

Target	Dilution	Host	Manufacturer	Catalog
ATF4	1:200	Rabbit	Proteintech (Rosemont, IL, USA)	10835-1-AP
CHOP	1:100	Rabbit	Novus Biologicals (Centennial, CO, USA)	NBP2-13172
CHOP	1:100	Mouse	Novus Biologicals (Centennial, CO, USA)	NB600-1335
Anti-Mouse AlexaFluor 488	1:500	Goat	Invitrogen (Waltham, MA, USA)	A-11029
Anti-Rabbit AlexaFluor 568	1:500	Goat	Invitrogen (Waltham, MA, USA)	A-11011
RBPMS	1:400	Rabbit	GeneTex (Irvine, CA, USA)	GTX118619
RBPMS	1:200	Mouse	Novus Biologicals (Centennial, CO, USA)	NBP2-03905
Beta-3-Tubulin	1:200	Rabbit	Abcam (Cambridge, UK)	ab18207
Beta-3-Tubulin	1:200	Mouse	Invitrogen (Waltham, MA, USA)	MA1-118

**Table 2:** Human donor pathology.

Tissue ID#	Ocular Disease	Age	Race	Sex	Cause of Death
600-10	Glaucoma treated w/drops and laser surgery	80	C	F	Acute cardiac crisis
608-10	Glaucoma	76	C	M	Lung cancer
616-10	Glaucoma, macular degeneration, cataract surgery both eyes	83	C	F	COPD
284-10	Glaucoma	67	C	M	Liver cancer
148-11	Glaucoma, cataract surgery OD (right)	71	C	M	Acute cardiac crisis
936-10	Glaucoma	82	C	M	COPD
1192-09	Normal; cataract surgery	96	C	F	GI Bleed
694-10	Normal	76	C	M	Congestive heart failure
236-11	Normal	74	C	M	Respiratory arrest
371-11	Normal	85	C	M	Probably heart disease
478-11	Normal	87	C	M	Probably myocardial infarction
997-10	Normal	88	C	M	Heart failure
1228-00	Normal; intraocular lens both eyes	83	C	M	Lung cancer; peptic ulcer disease

## Data Availability

The datasets used and/or analyzed in the present study are available from the corresponding author upon reasonable request.

## Supplementary Materials

The following supporting information can be downloaded at: https://www.mdpi.com/article/10.3390/ijms26051858/s1.
