# Supplementary material for: C/EBP Homologous Protein Expression in Retinal Ganglion Cells Induces Neurodegeneration in Mice
Source: Int J Mol Sci. 2025 Feb 21;26(5):1858. doi: 10.3390/ijms26051858 (PMC11899906; doi:10.3390/ijms26051858)
Supplement: Supplementary file 1 [file ijms-26-01858-s001.zip › ijms-3455066-supplementary.pdf]

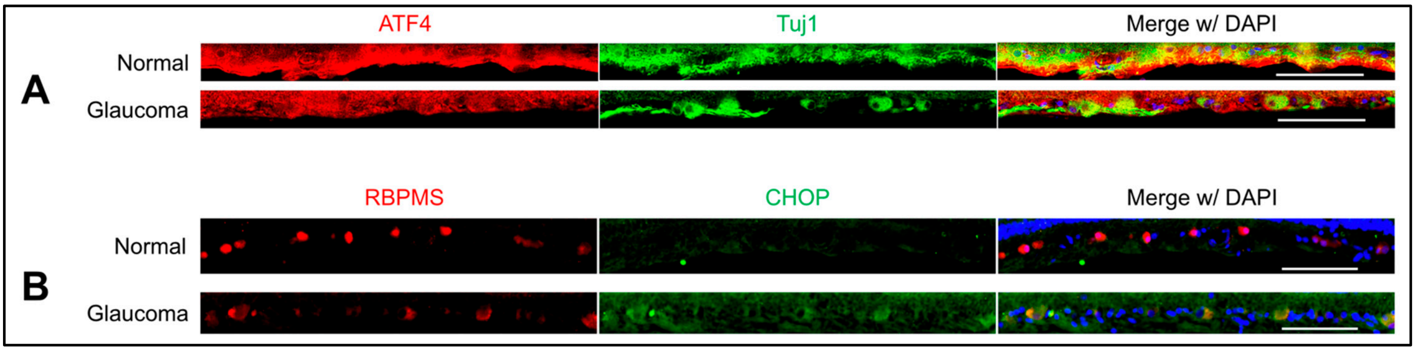

Figure S1. Cropped images of human IHC of **A)** ATF4 and Tuj1, as well as **B)** CHOP and RBPMS. Images were cropped to emphasize staining in the GCL. The scale bar is 100 μm.

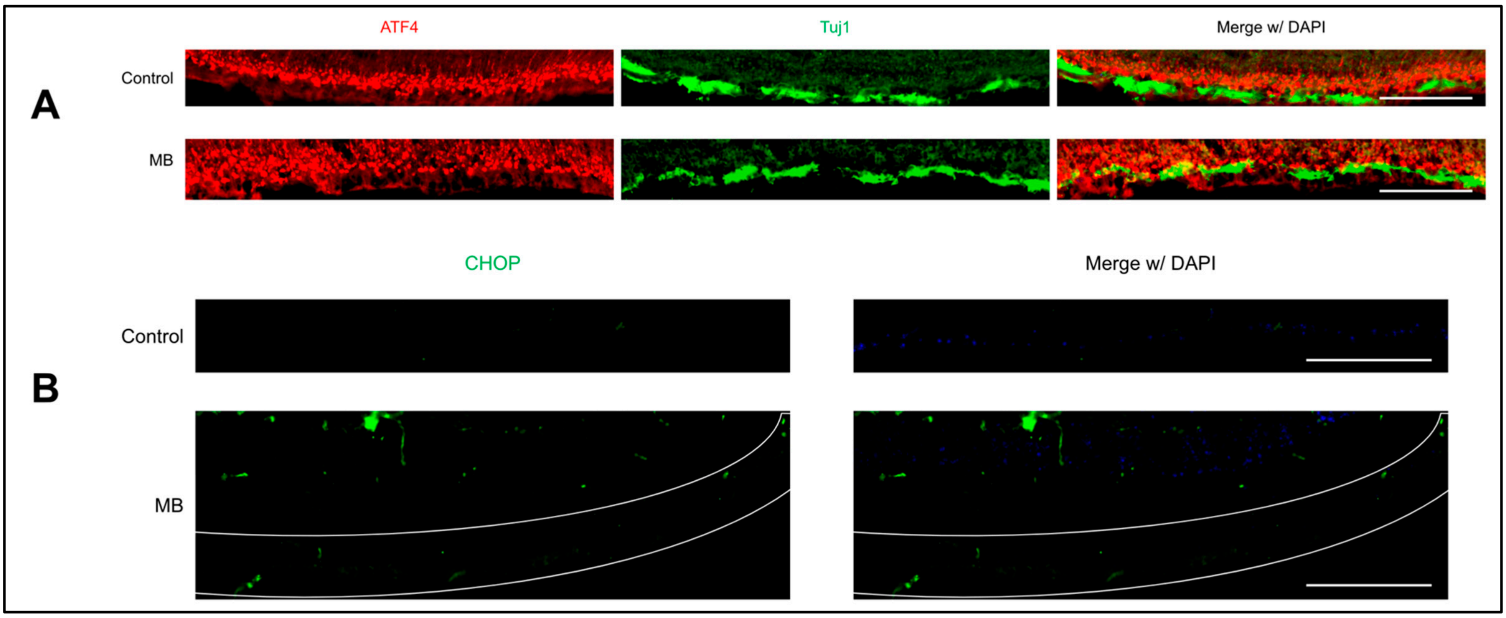

Figure S2. Cropped image of CHOP IHC staining in the mouse microbead model. Image was cropped to emphasize staining in the GCL. Due to the curvature of the retinal section, a white border was drawn around the GCL. The scale bar is 100 μm.

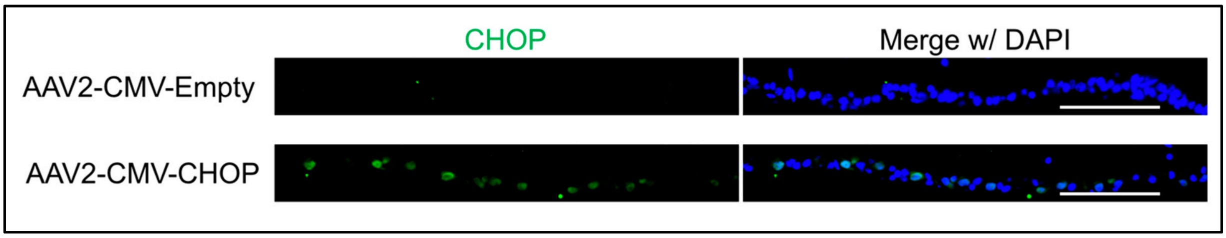

Figure S3. Cropped image of CHOP IHC staining in the mouse retinas after injection of AAV2-CMV-CHOP. Image was cropped to emphasize staining in the GCL. Due to the curvature of the retinal section, a white border was drawn around the GCL. The scale bar is 100 µm.

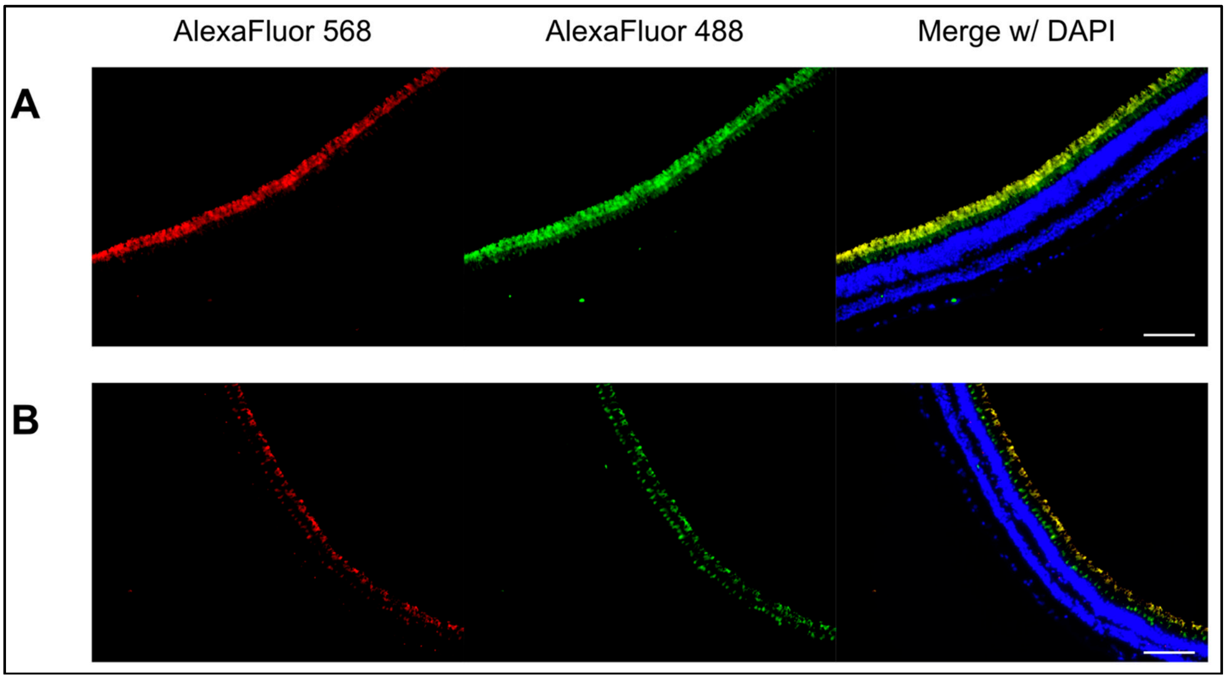

Figure S4. Primary antibody negative controls for **A)** ATF4/Tuj1 and **B)** CHOP/RBPMS immunofluorescent staining in human retinas. The scale bar is 100 µm.

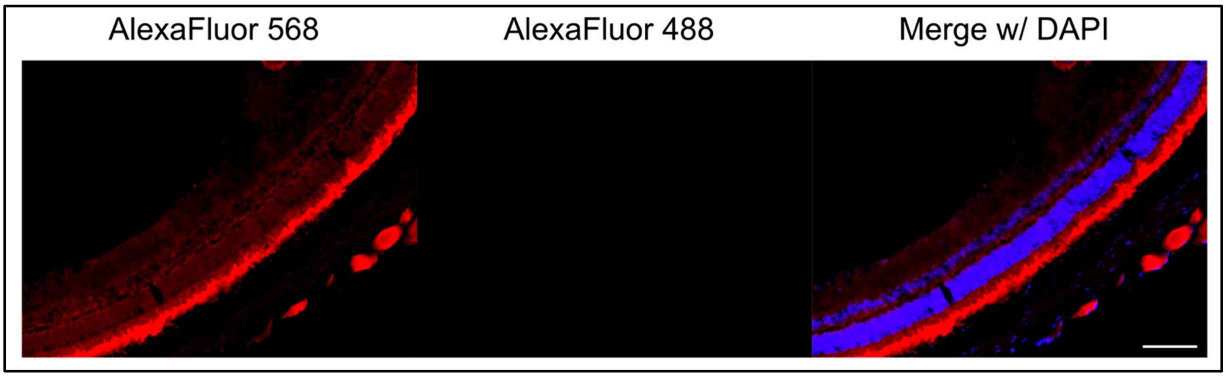

Figure S5. Primary antibody negative controls for retinal immunofluorescent staining in the magnetic microbead occlusion model. The scale bar is 100 μm.

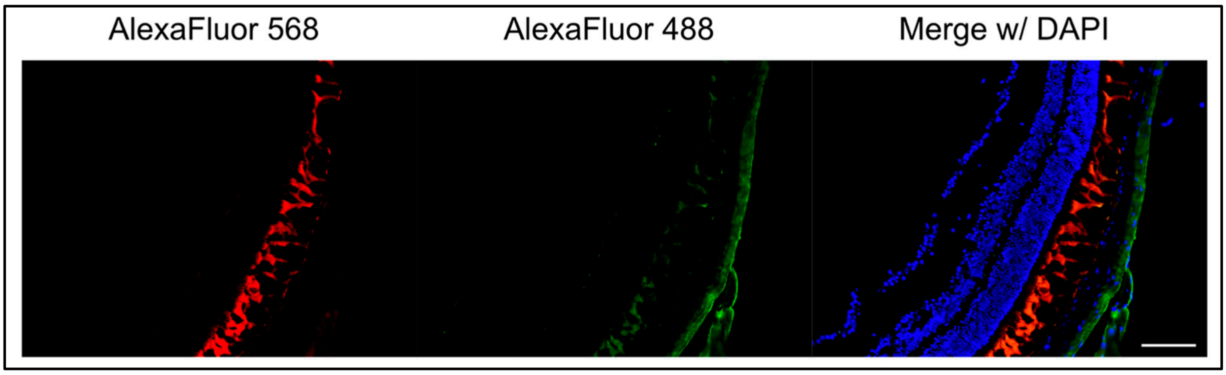

Figure S6. Primary antibody negative controls for retinal immunofluorescent staining in the AAV2-treated mice. The scale bar is 100 μm.
